# Endoscopic‐Assisted Spinal Approach to Remove a Broken Spinal Needle: Technical Notes and Case Report of a Complication of Spinal Anesthesia

**DOI:** 10.1155/cro/8123454

**Published:** 2026-04-15

**Authors:** Paolo Capitani, Matteo Messori, Pietro Domenico Giorgi, Giuseppe Rosario Schirò, Simona Legrenzi, Cristina Di Grigoli, Lorenzo Napoli, Valerio Moretti, Dario Capitani, Stefano Marco Paolo Rossi, Federico Bove, Mirko Poli

**Affiliations:** ^1^ Department of Orthopedic Surgery and Traumatology, ASST GOM Niguarda, Milan, Italy; ^2^ Residency Program in Orthopedics and Traumatology, University of Milan, Milan, Italy, unimi.it; ^3^ Department of Life Science Health and Health Professions, Università degli Studi Link Campus University, Rome, Italy

**Keywords:** arthroscopy, broken spinal needle, endoscopic-assisted spinal approach

## Abstract

**Background:**

Spinal anesthesia is widely used for many surgical procedures in orthopedic surgery. The breakage of a spinal needle within the patient′s intrathecal space represents a rare but potentially serious complication. The management of a broken spinal needle (BSN) remains unclear. Only a few clinical cases have been reported, and no surgical guidelines are available in the literature to date. Early surgical removal of the broken needle appears to be advisable.

**Case Presentation:**

A 65‐year‐old woman with a body mass index (BMI) of 32.1 kg/m^2^ was admitted to the operating theater for a knee arthroscopy. During spinal anesthesia, the tip of the needle broke off and remained in the patient′s back. The needle had a diameter of 25 gauge, and the retained fragment measured approximately 35 mm in length. Throughout the entire surgical procedure, the patient was maintained in the left lateral decubitus to prevent migration of the needle fragment. Initially, radiological landmarks were obtained with a C‐arm to localize the spinal needle. Once identified, a first percutaneous attempt to remove it with a different type of forceps was unsuccessful. Once endoscopic equipment was prepared, two small Farabeuf retractors and a self‐retaining retractor (Caspar lumbar retractor system) were positioned, and a 30° arthroscope was inserted by the surgeon. The broken needle was clearly visualized once arthroscopic saline inflow was initiated (inflow pump pressure 50 mmHg): the fluid dilated the muscle fibers and clearly exposed the BSN. The fragment was then successfully removed using arthroscopic grasping forceps, without any risk of mobilization or further breakage.

**Conclusions:**

In cases of a BSN during spinal anesthesia:

− early removal should be performed;
− the patient should be kept in the same position;
− radiological landmarks should be obtained using a C‐arm;
− if available, an endoscopic‐assisted spinal surgical approach should be considered as an effective and safe technique for needle removal.

## 1. Background

Spinal anesthesia is widely used for many surgical procedures in orthopedic surgery. It is known to offer several advantages, including less postoperative confusion, decreased need for strong analgesics, earlier resumption of oral intake, and less stress on cardiovascular and respiratory systems [1]. Nevertheless, spinal anesthesia is associated with a variety of complications that may occur with different frequencies. These can be mild, such as nausea and vomiting; moderate, such as hypotension, postdural puncture headache, and low‐frequency hearing loss; or severe, such as spinal hematoma, direct needle trauma, broken spinal needle (BSN), spinal cord injury, or spinal infection [2]. The breakage of a spinal needle within the patient′s intrathecal space represents one of the less common but potentially serious complications. This condition may cause neurological symptoms depending on the anatomical location of the fragment. Moreover, migration towards the spinal canal can result in nerve damage, leading to numbness, paresthesia, weakness, pain, infection, or cerebrospinal fluid leakage.

Currently, little is known about this complication. Abou‐Shameh et al. reported an incidence of 1:4000 spinal procedures performed in obstetric patients [3], whereas Martinello et al. reported only three cases of BSNs over the past 20 years during cesarean sections, with an approximate incidence of 1:11,000 [4]. A recent systematic review and meta‐analysis identified obesity, short stature, difficult anatomical landmarks, and small needle size as major risk factors for BSN [5]. Back and leg pain, followed by motor failure and paresthesia, have been reported as potential complications [6–8].

The management of BSN remains unclear. Only a few clinical cases have been reported, and no surgical guidelines are currently available in the literature. Early neurosurgical removal of a broken needle appears to be advisable, particularly when neurological symptoms develop over time [5]. However, apart from neurosurgical or general surgical approaches, no alternative techniques have been described.

Early endoscopic spine surgery was initially developed for the treatment of disc herniation as a less invasive and more precise alternative to traditional open techniques. Advances in technology and surgical expertise have expanded its application, allowing spine surgeons to address a wide range of spinal conditions using these techniques [9].

We report a case of a broken needle occurring during spinal anesthesia, which was successfully removed early using an endoscopic‐assisted spinal approach.

## 2. Case Presentation

A 65‐year‐old woman with a body mass index (BMI) of 32.1 kg/m^2^ was admitted to the operating theater for a knee arthroscopy. Magnetic resonance imaging revealed a complex tear of the posterior horn and body of the medial meniscus, as well as a tricompartmental chondropathy of the right knee. Informed consent for spinal anesthesia and right knee arthroscopy for meniscal treatment had been obtained. The patient was transferred to the operating theater and positioned on the operating table. Clinical examination using American Society of Anesthesiologists (ASA) standard monitoring revealed normal vital signs. The patient was then positioned in the left lateral decubitus for spinal anesthesia. Her past medical history was unremarkable for spinal disorders, previous lumbar surgery, or neurological deficits. Preoperative clinical evaluation, including ASA standard monitoring, revealed stable vital signs and no contraindications to spinal anesthesia.

The skin was sterilized, and local anesthesia was administered. During the spinal anesthesia procedure, the tip of the needle broke before administering the anesthesia and remained in the patient′s back, which was confirmed by fluoroscopy. The needle used (Sprotte Pajunk, Germany) was 25 gauge in diameter and 103 mm long. It had burr‐free, rounded, atraumatic edges, and no visible manufacturing defects prior to use.

An introducer was used, and the needle broke after more than five puncture attempts. The needle broke during insertion without external bending or angulation maneuver. The retained fragment of the broken needle measured approximately 35 mm in length.

The patient was promptly informed about the complication, and a spine surgeon was consulted to plan the removal of the needle. At that time, the patient was asymptomatic and showed no neurological deficits. The decision‐making process considered three possible management strategies: (1) conservative management with radiological follow‐up, (2) delayed open surgical removal, or (3) immediate minimally invasive retrieval. Given the potential risk of fragment migration towards the spinal canal, the fragment length (~35 mm), the relatively superficial location, and the availability of spine surgical expertise, early surgical removal was considered the safest option.

Importantly, the left lateral decubitus was maintained throughout the surgical procedure to avoid spinal needle mobilization.

**Figure 1:**
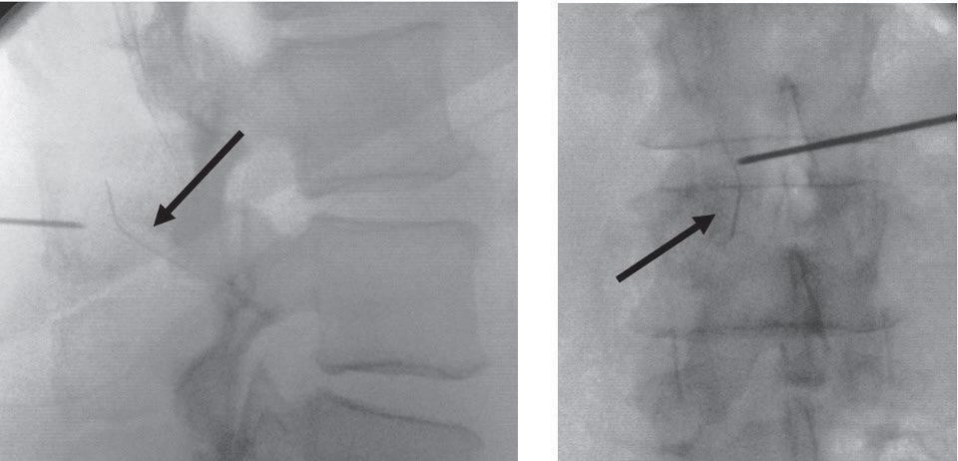
Anteroposterior and lateral x‐rays of lumbar spine immediately after broken spinal needle, during centering with a metal marker and C‐arm. Black arrows indicate spinal needle in the erector spinae muscles.

**Figure 2:**
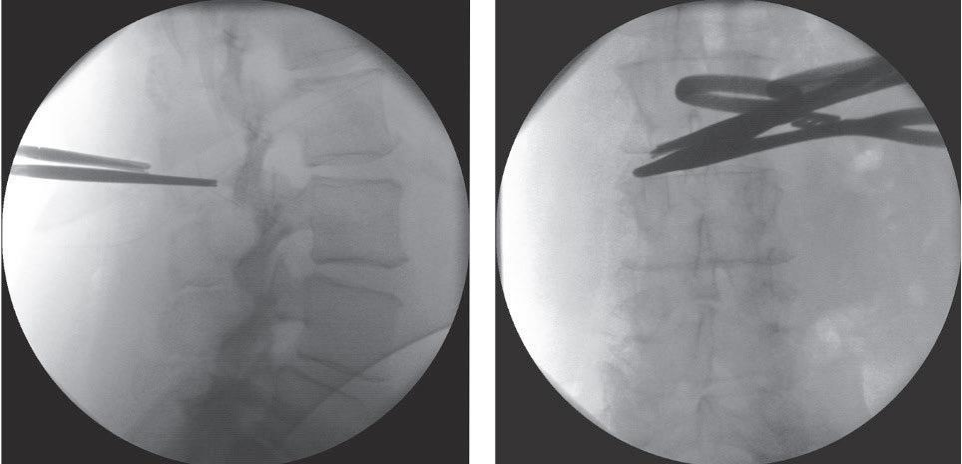
Anteroposterior and lateral x‐rays of the lumbar spine during the initial attempts to remove the broken spinal needle.

The retrieval procedure was started 20 min after the BSN discovery. A general anesthesia was chosen for the needle retrieval procedure. A sterile field was made. First, radiological landmarks were obtained using a C‐arm to identify the position of the spinal needle. The fragment was located within the erector spinae muscle at a depth of approximately 6 cm from the skin, 1 cm to the left of the L2 spinous process (Figure 1). An initial percutaneous attempt using different grasping forceps under fluoroscopic guidance was unsuccessful due to limited visualization and insufficient control of the fragment axis. (Figure 2).

Subsequently, a 3‐cm left paramedian longitudinal incision was performed, and a second open attempt was planned. Blunt muscle splitting was carried out to approach the posterior extremity of the fragment, which became palpable. However, repeated attempts at blind extraction with forceps were unsuccessful and were judged potentially unsafe due to the risk of further fragment mobilization or breakage.

**Figure 3:**
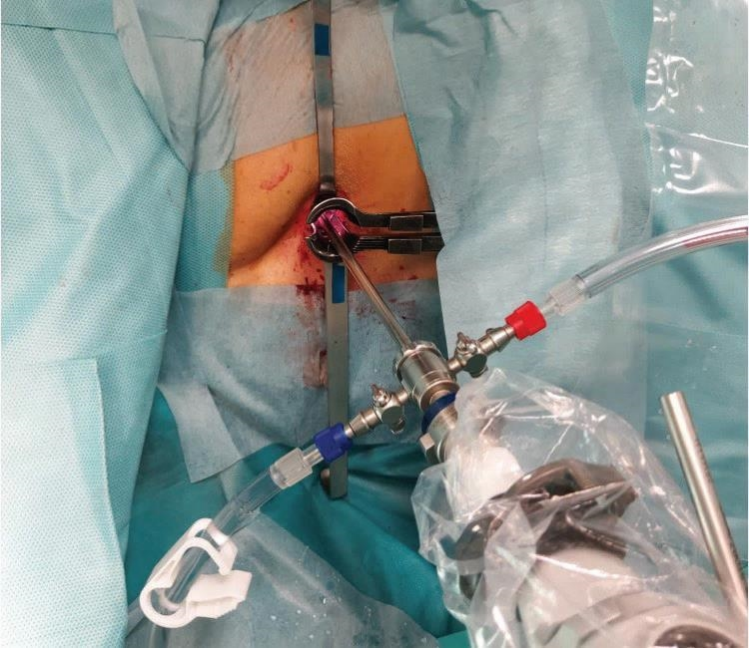
Minimally invasive spinal approach endoscopic‐assisted with Caspar lumbar retractor system.

At this stage, an endoscopic‐assisted approach was adopted. Two small Farabeuf retractors and a Caspar lumbar retractor system were positioned to create a minimally invasive working corridor. A 30° arthroscope was introduced into the operative field. (Figure 3). It should be emphasized that the working space created by the Caspar lumbar retractor system is relatively narrow. Therefore, progressive muscle dilation is required to obtain an adequate operative corridor. In our experience, achieving approximately 20°–30° of instrumental maneuverability is essential to safely align the grasping forceps with the longitudinal axis of the needle fragment. Insufficient angular freedom may increase the risk of fragment mobilization or further breakage. For this reason, careful widening of the working space and continuous endoscopic visualization are mandatory during fragment extraction.

Arthroscopic saline irrigation was initiated with an inflow pressure of 50 mmHg. The hydrostatic effect allowed controlled separation of muscle fibers and improved visualization of the retained needle fragment without excessive tissue dissection. Careful monitoring of irrigation pressure was maintained throughout the procedure to avoid potential epidural pressure transmission.

Under direct endoscopic visualization, the needle fragment was clearly identified and aligned with an arthroscopic grasping forceps. The needle fragment was then removed using an arthroscopic grasping forceps, without risk of mobilization or further breakage (Figures 4 and 5). Fluoroscopic imaging confirmed complete removal of the needle. The skin was closed using a nonabsorbable 2.0 suture. The removal procedure takes approximately 45 min. No neurological symptoms or cerebrospinal fluid leakages were observed postoperatively. The planned arthroscopic surgery of the right knee was subsequently performed without complications.

Figure 4(a) Endoscopic view of the broken spinal needle and (b) lateral x‐ray of spinal arthroscopy setup with the retractors.

**Figure:**
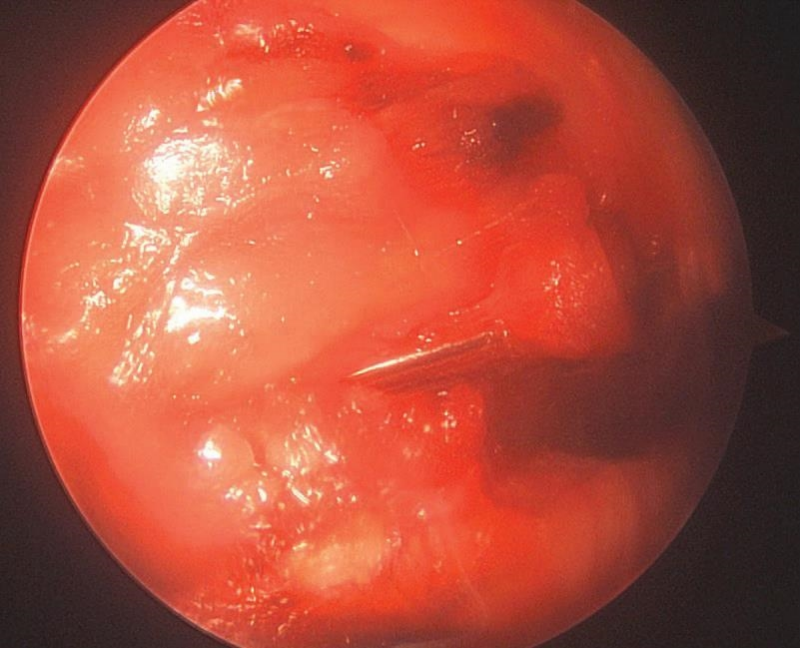
(a)

**Figure:**
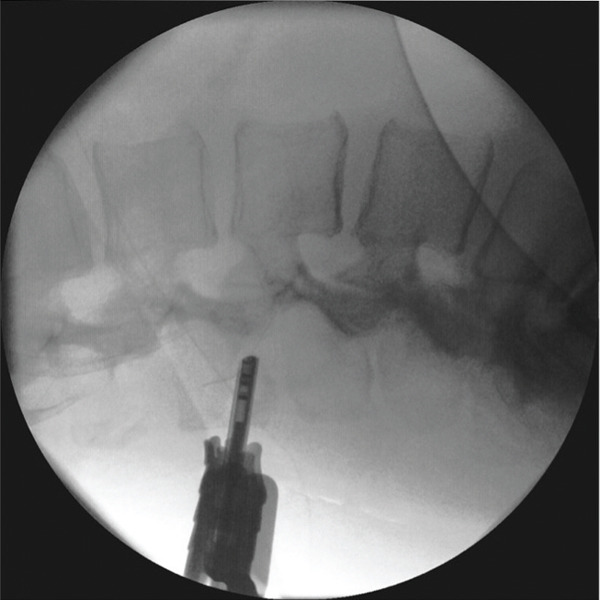
(b)

Figure 5The arthroscopic grasping forceps used (a) to remove the broken spinal needle (b).

**Figure:**
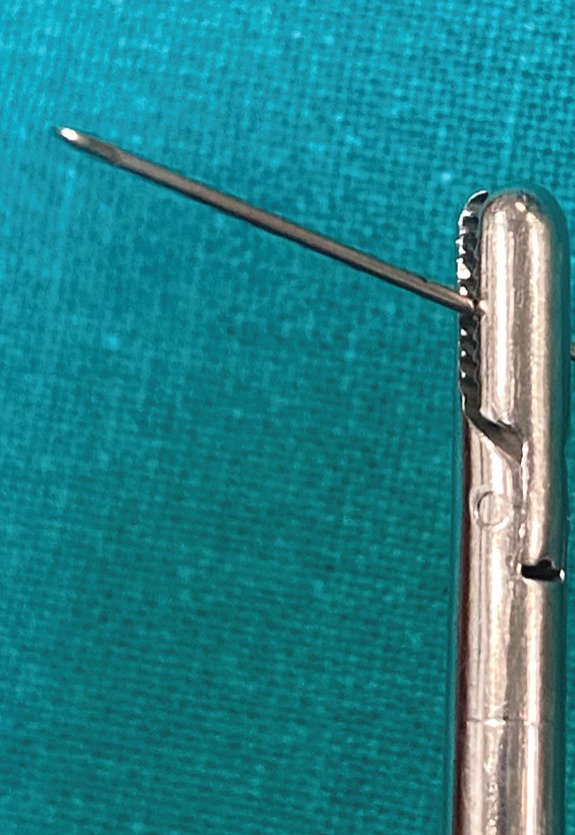
(a)

**Figure:**
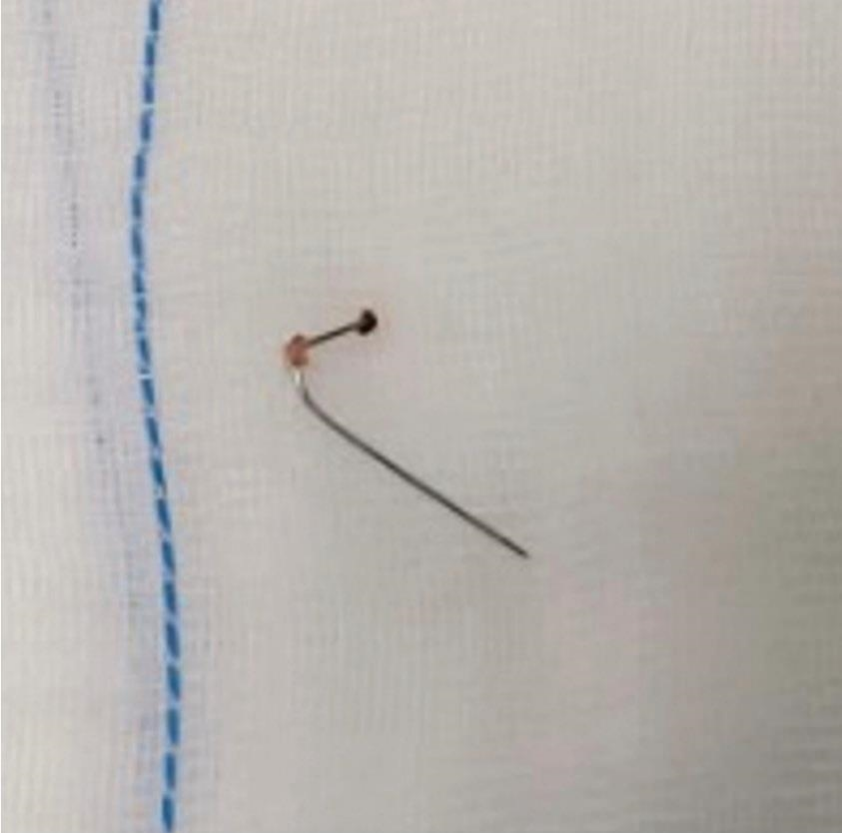
(b)

On postoperative Day 1, lumbar incision pain was rated as 1/10 on the Visual Analog Scale (VAS) without analgesic requirement. Knee pain was rated 4/10 and managed routinely.

The patient was followed clinically at 1, 6, and 12 months, and annually thereafter. At 5‐year follow‐up, she did not report lumbar pain, sciatic pain, radicular symptoms, or any acute or chronic local discomfort at the surgical site.

Neurological examination remained normal throughout follow‐up. The patient resumed all daily and recreational activities without limitations. No delayed complications, such as infection, foreign body reaction, or mechanical low back pain, were observed.

From the patient′s perspective, she reported initial anxiety related to the intraoperative complication but expressed satisfaction with the transparency of communication and the immediate management strategy. At long‐term follow‐up, she reported no residual concerns related to the event.

Regarding device failure analysis, no visible structural defect was identified in the remaining proximal needle segment. The breakage occurred after multiple puncture attempts in a patient with increased BMI and potentially difficult anatomical landmarks. Small‐gauge needles are known to be more susceptible to deflection and mechanical stress. Although no excessive bending maneuver was reported, repeated redirection within dense paraspinal tissues may have contributed to structural fatigue and subsequent fracture. No formal manufacturer analysis was performed, which represents a limitation.

## 3. Discussion

There is a lack of consensus in the literature regarding the surgical management of foreign bodies in the back. Among the potential complications of spinal anesthesia, spinal needle breakage occurs infrequently but remains a serious issue, burdened by a series of potentially adverse effects. Although its incidence and risk factors have been analyzed over the past decades, little is known about its surgical management. As reported by Alsharif et al. in their 2023 systemic review [5], a number of potential risk factors have been hypothesized, both patient‐ and instrument‐related. Firstly, the difficult determination of anatomical landmarks, as is often the case in obese and pregnant patients, poses a critical challenge for the practitioner. As noted by Taylor et al. [10], higher BMI patients may require additional precautions during spinal anesthesia, whereas Atashkhoei et al. [11] point out that lower BMI patients may have a relatively lower risk of needle breakage. This highlights the potential benefit of ultrasound guidance, which could serve as a valid tool for guiding needle placement in morbidly obese patients [12, 13]. Moreover, it has been suggested that the lower the needle diameter, the higher the risk of breakage, given that the majority of the studies analyzed involved the use of 25 gauge needles or lower [5]. Smaller gauge needles are reported to correlate with greater deflection in in vitro studies [14], although they are typically chosen to minimize the incidence of postdural puncture headache [15, 16].

Despite this, there are no technical notes or practical tips in the literature on the surgical management of the removal of a BSN.

In the majority of case reports, spinal needles were successfully removed immediately after their breakage. Alsharif et al. [17] performed a paramedian incision with muscles splitting using intraoperative fluoroscopy guidance in both the anteroposterior and lateral views to extract the needle, as also shown in other reports [18, 19]. However, in several cases, it was reported that the BSN remained in place for several days. On average, needles were removed within 1–4 days; although Kabore et al. [20] planned the removal of the needle under general anesthesia by a neurosurgeon on the 13th postoperative day, after educating the patient about any warning signs. Moreover, in a small number of case reports, the BSN was discovered several months later [6], or even left in situ, causing neurological symptoms 3 years later after migrating closer to spinal roots [21]. In all these cases, surgical extraction through a minimally invasive spinal surgery was described, using a lumbar paramedian approach at the correct intervertebral space [22]. In these procedures, patients were placed in a prone position, and a posterior surgical incision of at least 5–6 cm was made to adequately expose muscle tissue for spinal needle removal.

In our case, we demonstrate that a safe surgical removal of a BSN can be achieved maintaining the lateral decubitus position (the same used for anesthesia, without moving the patient) through an endoscopic‐assisted minimally invasive spinal approach.

In open spinal surgery, the space is created by clearing the soft tissue, including muscle, through various retraction movements.

In literature, just a few case reports describe the removal of foreign bodies, such as bullets, with an endoscopic technique [23, 24].

The goal of these techniques is to remove the foreign body while minimising the comorbidities associated with surgery. Endoscopic‐assisted paramedian lumbar surgery helps identifying the spinal needle easily without requiring more aggressive surgical solutions. An extended open surgical approach would have facilitated identification of the broken needle, but would have led to extensive muscle disconnection with consequent increased pain at the incision site and potential subsequent functional limitation and delayed recovery. In addition to the increased risk of infection resulting from a larger surgical incision.

In order to prevent fluid‐related complications in the spinal approach, it is important to understand the anatomical differences between the spine and a joint. The working space is very close to the epidural space, without any separating structure (such as a joint capsule). For this reason, careful control of saline pressure is required to avoid negative effects on the neural system during endoscopic spinal surgery. To minimize the risk of complications in fluid‐medium spinal surgery, control of pressure inside the working space is recommended: the optimal hydrostatic pressure is 30–50 mmHg [25].

According to the literature, less tension is required to remove spinal catheter when the patient is in lateral decubitus as opposed to a sitting position [26, 27]. Additionally, significantly less force appears to be required to remove a BSN when the patient maintains the same position as at the time of insertion [28]. Moreover, we strongly believe that using minimally invasive surgery endoscopic‐assisted might reduce risks of broken needle mobilization and it involves low postoperative pain caused by the surgical approach. If removal of BSN is required, we recommend an endoscopic‐assisted minimally invasive spinal approach as a valid surgical technique.

Our study has numerous limitations. Firstly, the absence of a control group or comparative analysis in a case report limits our ability to assess the superiority of the endoscopic approach over other methods, such as open surgery or neurosurgical techniques.

## 4. Conclusions

Spinal anesthesia is commonly used in orthopedic surgery. One of the most dangerous complications of spinal anesthesia is BSN. If it happens:

− remove it as early as possible;
− keep the patient in the same position;
− obtain radiological landmarks using a c‐arm;
− if available, an endoscopic‐assisted spinal surgical approach should be considered, as it is an effective procedure that allows safe removal, given a substantial limitation: the technique requires anyway specialized endoscopic equipment and expertise, which may not be available in all hospitals, particularly in low‐resource settings.

## Funding

Open access publishing facilitated by Azienda Socio Sanitaria Territoriale Grande Ospedale Metropolitano Niguarda, as part of the Wiley – SBBL agreement.

## Ethics Statement

This study was conducted in accordance with EN ISO 14155: 1, EN ISO 14155: 2, Declaration of Helsinki, and Good Clinical Practices (GCP) guidelines.

## Consent

Written informed consent was obtained from the patient (and/or his/her legal guardians) for treatment and for publication of this case report. No ethical approval was applicable, as the data were collected as part of routine clinical care.

## Conflicts of Interest

The authors declare no conflicts of interest.

## Data Availability

Research data are not shared.
